# Lipid Nanoparticles as Carriers for Bioactive Delivery

**DOI:** 10.3389/fchem.2021.580118

**Published:** 2021-04-23

**Authors:** Neerupma Dhiman, Rajendra Awasthi, Bhupesh Sharma, Harsha Kharkwal, Giriraj T. Kulkarni

**Affiliations:** ^1^Amity Institute of Pharmacy, Amity University Uttar Pradesh, Noida, India; ^2^Amity Institute of Phytomedicine and Phytochemistry, Amity University Uttar Pradesh, Noida, India

**Keywords:** solid lipid nanoparticles, nanostructure lipid carriers, toxicity, scale up, stability, recent advances

## Abstract

Nanotechnology has made a great impact on the pharmaceutical, biotechnology, food, and cosmetics industries. More than 40% of the approved drugs are lipophilic and have poor solubility. This is the major rate-limiting step that influences the release profile and bioavailability of drugs. Several approaches have been reported to administer lipophilic drugs with improved solubility and bioavailability. Nanotechnology plays a crucial role in the targeted delivery of poorly soluble drugs. Nanotechnology-based drug delivery systems can be classified as solid lipid nanoparticulate drug delivery systems, emulsion-based nanodrug delivery systems, vesicular drug delivery systems, etc. Nanotechnology presents a new frontier in research and development to conquer the limitations coupled with the conventional drug delivery systems through the formation of specific functionalized particles. This review presents a bird's eye view on various aspects of lipid nanoparticles as carriers of bioactive molecules that is, synthesis, characterization, advantage, disadvantage, toxicity, and application in the medical field. Update on recent development in terms of patents and clinical trials of solid lipid nanoparticles (SLNs) and nanostructure lipid carriers (NLCs) have also been discussed in this article.

## Introduction

The main objective of designing an effective drug delivery system is to deliver the therapeutic molecules to target site by minimizing premature drug degradation, maintain optimum amount of drug at the tissue of interest to produce better therapeutic outcomes, and to prevent harmful side effects. The conventional methods of drug administration have several limitations, such as increased chances of dose missing, fluctuations in drug levels, poor bioavailability, undesirable side effects, poor patient compliance, rapid metabolism, and drug toxicity. However, these limitations can be overcome by using target-specific nanocarrier systems, such as solid lipid nanoparticles (SLNs), niosomes, liposomes, ethosomes, bilosomes, transferosomes, colloidosomes, pharmacosomes, herbosomes, layerosomes, sphingosomes, ufosomes, and polymeric nanoparticles. Since the disclosure of cosmetic applications of non-ionic surfactant vesicles by L'Oreal in 1975, the development of nanomaterials for drug delivery and biomedical applications is growing exponentially (Awasthi et al., 2014). Nanocarriers have several advantages, such as enhanced solubility and bioavailability of hydrophobic molecules, reduce dose by controlled and sustained release of therapeutic agents, and attenuate drug toxicity. Nanoparticles are gaining significant attention of formulation scientists in drug delivery due to their unique physicochemical properties, ultra-small particle size, and ability to cross various biological barriers (Pandey et al., 2015; Singh et al., 2016). Nanoparticles are nanometer-sized (10–1,000 nm) particles that encapsulate therapeutic agents within the particle-matrix, adsorbed or conjugated through functional modifications onto the surface (Limayem et al., 2004; Garg et al., 2014; Singh et al., 2016; Awasthi et al., 2018; Maurya et al., 2019). The nanoparticulate drug delivery systems comprise two components: a drug and its carrier. Several inorganic and organic carriers have been reported that are selected based on the drug properties and desired therapeutic action. These nanocarriers can protect the drug molecules against hydrolytic and enzymatic degradation, prolong circulation time, and ameliorate its therapeutic benefits (Awasthi et al., 2020).

**Figure 1:**
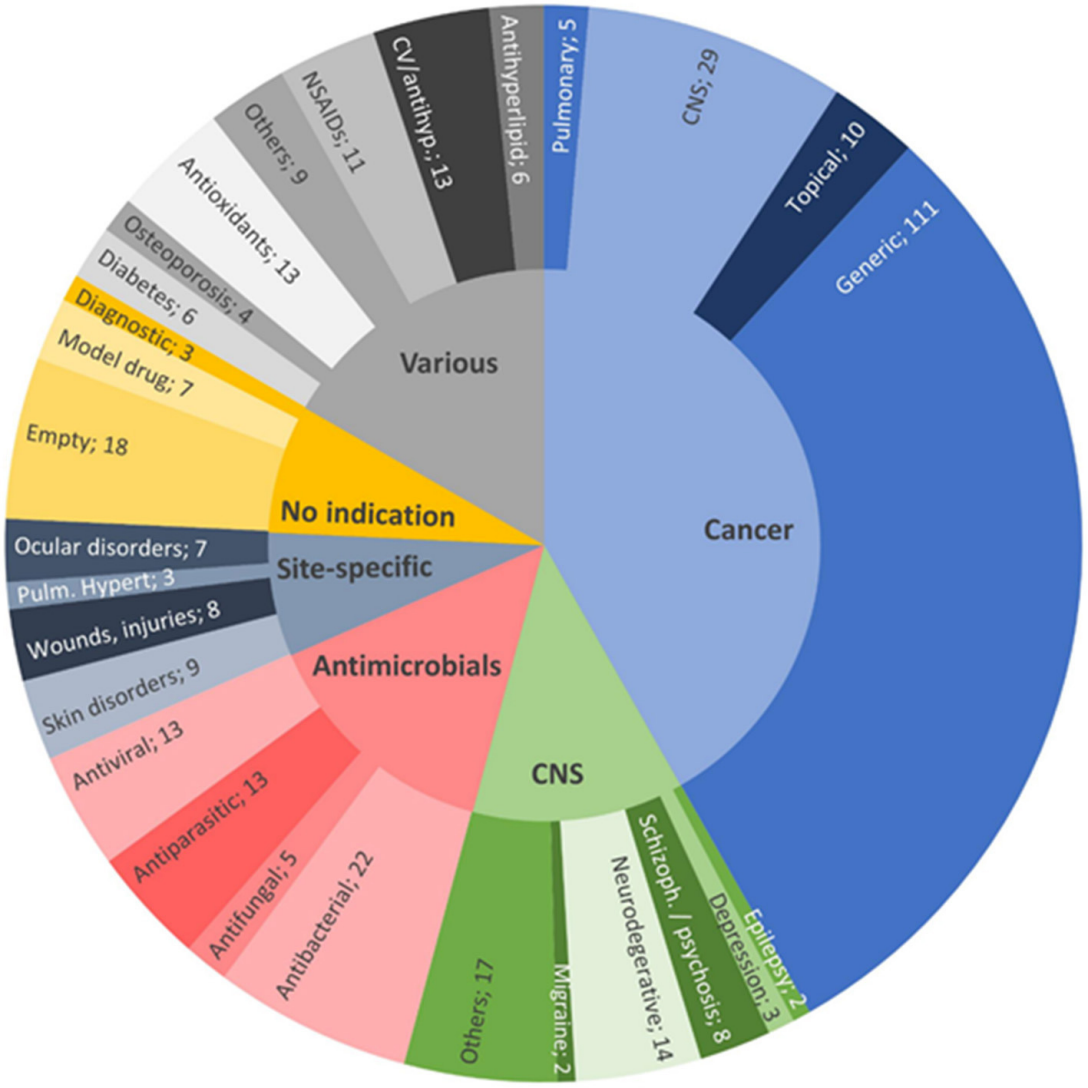
Distribution of the reviewed publications between 2013 and 2020 on NLCs and SLNs, by therapeutic field: anticancer therapies (41.8%, light blue); various indications (16.7%, gray); antimicrobials (14.3%, pink); CNS diseases, excluding cancer and infection (12.4%, green); nanovehicles not intended for any specific therapeutic area (7.5%, yellow); and site-specific treatments (7.3%, dark blue). Reproduced with permission from Frontiers in Molecular Biosciences (Montoto et al., 2020).

Despite the huge burden of major health issues, there is a lack of effective therapeutic approaches that could control the onset and/or disease progression of disease. Pharmaceutical scientists are exploring new techniques to enhance the therapeutic benefits of drugs. During the past two to three decades, lipid-based nanocarriers for bioactive delivery have gained significant attention of researchers from both academia and industries. Publication statistics of scientific papers, patents, and clinical trials on various aspects of these nanocarriers indicate that the interest in the field of development of lipid-based nanocarriers remains high. Figure 1 presents publication statistics of scientific papers published during 2013 to 2020 on nanostructured lipid carriers (NLCs) and SLNs in various therapeutic areas (Montoto et al., 2020). These nanocarriers increase the solubility of lipophilic drugs which will augment permeability and hence improve the bioavailability of the drug. The basis for the selection of SLNs and NLCs is that they are easily amendable, safe, and can be prolonged by regulating the release of loaded drugs.

In this review, we first recapitulated a brief introduction, common ingredients, properties, benefits, and drawbacks of SLNs. Second, we discussed types of NLCs and formulation approaches used for the preparation of SLNs and NLCs. Finally, we outlined characterization, scale-up and stability issues, basic applications in drug delivery, and toxicity issues of these systems in disease treatments.

## Lipid Nanoparticulate Drug Delivery Systems

**Figure 2:**
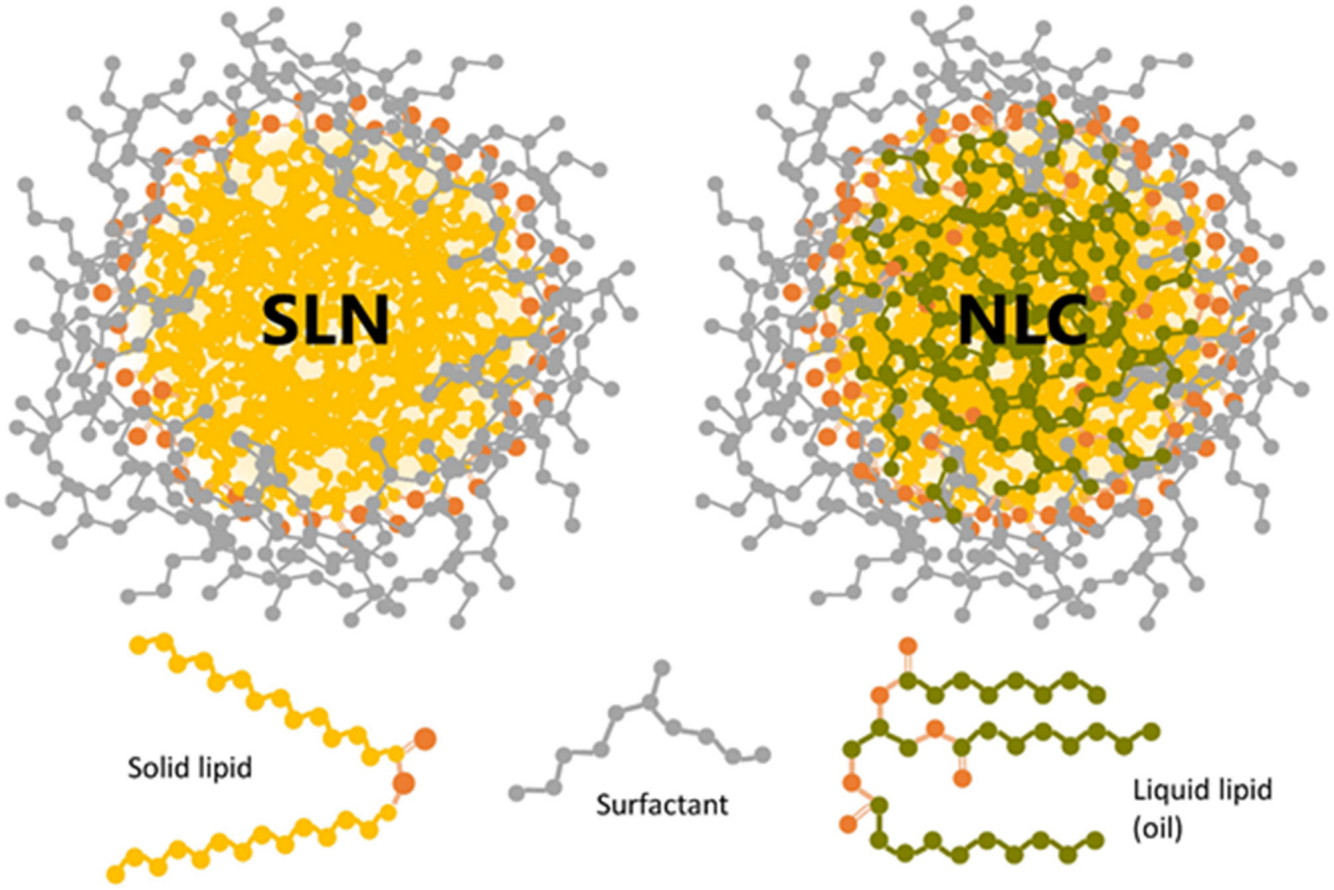
Schematic representation of sterically stabilized NLCs and SLNs with a neutral surfactant. The oxygen atoms in solid and liquid lipids are shown in orange color. Reproduced with permission from Frontiers in Molecular Biosciences (Montoto et al., 2020).

Due to the use of biocompatible and biodegradable lipids, these lipid-based nanocarriers have gained tremendous interest during the past two decades. Lipid nanocarriers are preferred over polymeric nanoparticles. Lipid nanoparticles can resolve the challenges associated with polymeric nanoparticles, such as cytotoxicity and lack of suitable methods for large-scale production (Haider et al., 2020). The lipid nanocarriers can be categorized into liposomes, niosomes, SLNs, and NLCs. Schematic representation of sterically stabilized NLCs and SLNs with a neutral surfactant is shown in Figure 2 (Scioli Montoto et al., 2020). SLNs and NLCs overcome the limitations of the conventional colloidal carrier systems, such as liposomes, niosomes, nanoemulsions, and polymeric nanoparticles (Weber et al., 2014). Lipid nanoparticles can substantially improve the solubility, bioavailability, pharmacokinetic parameters, intestinal absorption, skin penetrability, and ocular residence time of drugs which helps the molecule to cross the physiological barriers and decrease its side effects. Thus, these drug delivery carriers demonstrate significant potential in pharmaceutical or medical applications. The lipid particulate drug delivery system can be classified into SLNs and NLCs. It has been reported that the drug release is slower in SLNs than in NLCs at low drug-loading. However, at high drug-loading, no significant difference in drug release from SLNs and NLCs is observed. NLCs are more stable than SLNs at 25°C (Das et al., 2012).

### Solid Lipid Nanoparticles

**Figure 3:**
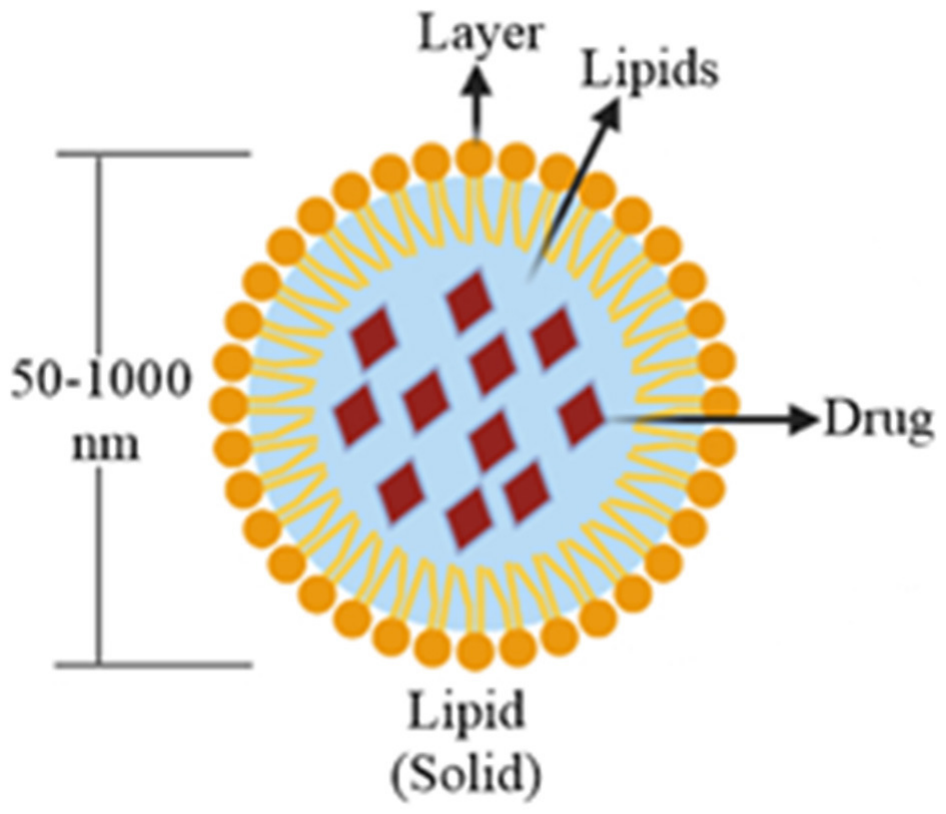
Structure of drug-loaded SLN particle.

Due to their compatibility and penetration through different physiological barriers, these nanocarriers (50–1,000 nm) have gained the tremendous interest of formulation scientists during the past two decades. Solid lipids are gaining popularity over the liquid oils in the preparation of controlled release of nanoformulations due to the low mobility of drug molecules in a solid lipid matrix (Mehnert and Mäder, 2012). These particles are formed by enclosing a solid lipid core into a lipid monolayer (Kammari et al., 2017) (Figure 3). SLNs are useful in the area of clinical research (Singh et al., 2012), cosmetics and pharmaceuticals (Garg et al., 2012), and in research (Garud et al., 2012). The use of biological lipids in the fabrication of SLNs reducesthe possibility of acute or chronic toxicities (Weber et al., 2014). SLNs with a particle size of 120–200 nm can bypass the liver and spleen filtration. SLNs have several advantages over other nanocarriers, such as their easy large-scale production, increased bioavailability, low chronic, or acute toxicit; incorporation of both hydrophilic and lipophilic drugs can be done. These properties of SLNs make them ideal drug carrier over other nanoformulations (Srivastava et al., 2021).

#### Ingredients/Components of SLNs

**Figure 4:**
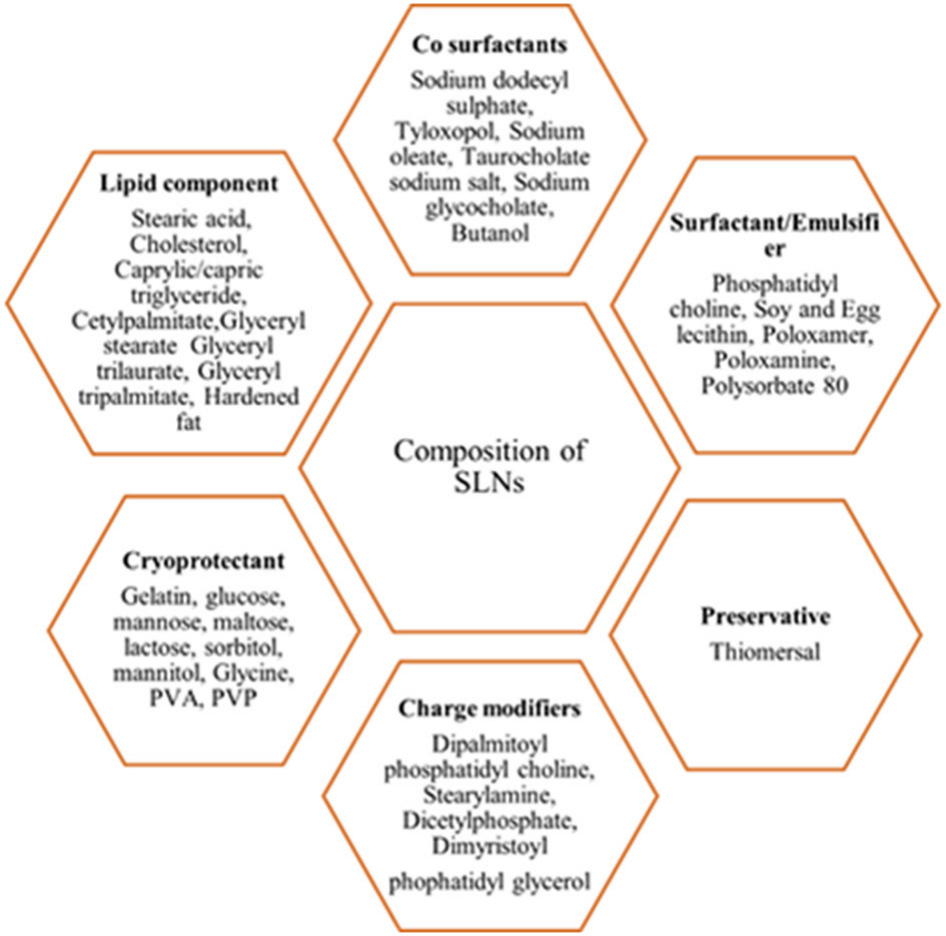
Composition of the SLNs.

SLNs are comprised of two basic components, such as the matrix materials (lipids) and the surface stabilizers; other components include co-surfactant, preservatives, cryoprotectant, and charge modifiers (Figure 4) (Mishra et al., 2018). The matrix material constitutes of lipids, such as monoacid triglycerides (tristearin, tripalmitin, and trilaurin), fatty acids (stearic acid), steroids (cholesterol), waxes (cetylpalmitate), partial glycerides (imwitor), fats, and waxes. The preferred surface stabilizers used include phospholipids, bile salts, soyabean lecithin, egg lecithin, phosphatidylcholine, poloxamers, and polysorbates (Mehnert and Mäder, 2012; Mishra et al., 2018). The central solid-lipid core of SLNs solubilizes hydrophobic molecules in the presence of suitable surfactants (Mishra et al., 2018).

#### Advantages of SLNs in Drug Delivery

Drug administration using SLN-based drug carriers appear to be an attractive approach for several reasons including:

- The SLNs are crucial in the site-specific targeting of the drug and improve the stability of the drug incorporated into it (Seyfoddin et al., 2010; Singh et al., 2016).
- The SLNs have increased drug-loading efficiency or high percentage encapsulation efficiency (Joshi et al., 2014; Singh et al., 2016).
- The SLN core has higher entrapment efficiency for hydrophobic drugs than liposomes (Kammari et al., 2017).
- Capability to encapsulate hydrophobic as well as hydrophilic drug substances (Kaur et al., 2015; Singh et al., 2016). But lipophilic drugs are the drugs of choice which are encapsulated in SLNs (Garud et al., 2012).
- As the preparation of the SLNs does not require the organic solvent in the preparation method, these are free from organic solvents (Liu et al., 2009; Singh et al., 2016).
- The SLNs have the lipid matrix which will entrap the drug in it and hence increases the bioavailability of the compound (Goyal et al., 2014; Singh et al., 2016).
- The lipids used in the preparation of the matrix are non-toxic (Dong et al., 2011; Singh et al., 2016).
- Surface functionality of the system can be modified by altering the particle size and surface charge (Awasthi et al., 2016).
- The production yield is high, and the process scale-up is easy than other types of nanoparticles.
- SLNs have been reported for the administration *via* parenteral (Wissing et al., 2004), oral, topical (Puglia et al., 2008) pulmonary (Liu et al., 2008), ocular (Liu et al., 2012), and rectal (Sznitowska et al., 2001) routes.
- SLNs control the release profile and improve the stability of encapsulated drug molecules (Garg et al., 2013; Weber et al., 2014).

#### Disadvantages of SLNs in Drug Delivery

Despite several benefits, the drugs administered *via* SLNs have few limitations, such as:

- Crystalline lipids may minimize the drug loading capacity.
- Sometimes the β-modification of the crystalline lipids causes the leaking of the encapsulated drug.
- Gelling of the dispersed phase takes place during storage.
- A high content of water (70–90%) may lead to stability problems.
- The loading of the drug in the SLNs depends on various factors, such as the interaction of the drug with the lipid melt, nature of lipid matrix, drug miscibility with lipid matrix, and the drug being distributed or dissolved in the lipid matrix (Mishra et al., 2018).

### Nanostructure Lipid Carriers

**Figure 5:**
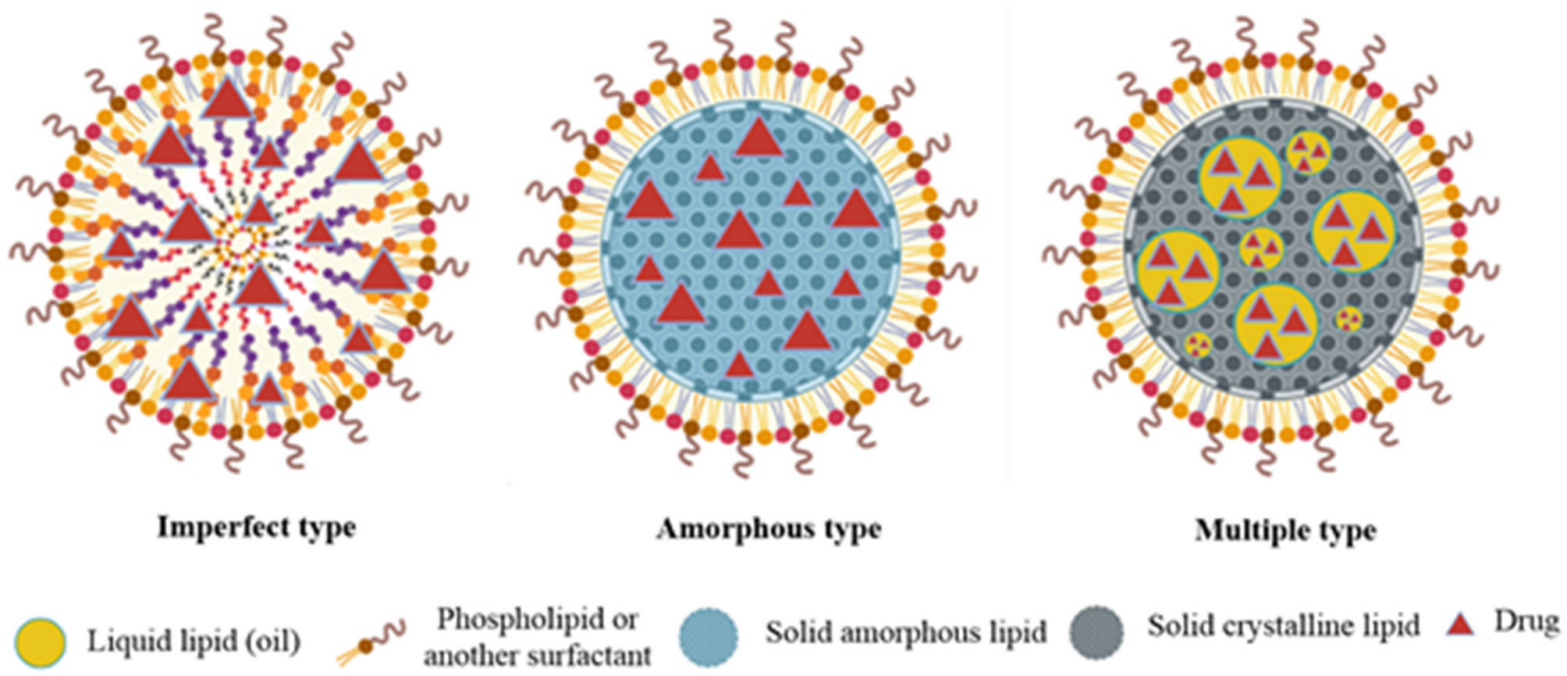
Diagrammatic presentation of imperfect, amorphous, and multiple type NLCs.

Nanostructure lipid carriers are designed to overcome the limitations of SLNs and are hence known as the second generation of SLNs (Wang et al., 2013). These colloidal drug carrier systems offer targeted delivery of drugs (Luan et al., 2013) and increase the bioavailability of hydrophobic drugs, and protect sensitive active compounds (Wang and Xia, 2014). NLCs, like SLNs, exist as a solid matrix of lipids at room and body temperature but they have different internal structures as compared to the SLNs. In NLC formulations, a portion of solid lipid is replaced by oil resulting in a less, ordered lipid matrix which improves the drug loading efficiency and prevents drug leaching and oxidation of the drug during storage. NLCs are classified depending on the lipid and oil mixture composition (Weber et al., 2014). Three different types of NLCs include (i) imperfect type, (ii) amorphous type, and (iii) multiple oil-in-solid fat-in-water type (Figure 5).

#### The Imperfect Type

The imperfect NLCs are prepared by mixing structurally different lipids which can distort crystal order. This distortion can be enhanced by changing the saturation and number of carbon atoms in lipids. This leads to an increase in the loading capacity (Khosa et al., 2018).

#### The Amorphous Type

In amorphous type NLCs, an amorphous matrix is formed by mixing lipids, such as hydroxy octacosanyl hydroxystearate or iso-propyl myristate with a solid lipid. Due to these materials, the NLCs exist in an amorphous state which prevents drug expulsion resulting due to the β-modification during storage (Khosa et al., 2018).

#### Multiple Oil-in-Solid Fat-in-Water Type

These NLCs have the advantage of increased drug loading and prolonged release of drugs. This is due to the presence of abundant nanosized oil droplets dispersed in the solid matrix (Muller et al., 2002).

### Preparation Methods for SLNs and NLCs

Several methods, such as high shear homogenization, high-pressure homogenization (cold and hot homogenization), solvent evaporation/emulsification, etc. have been reported to produce SLNs. Solid lipid component of SLNs may contain ingredients, such as partial glycerides (imwitor), triglycerides (tristearin), fatty acids (stearic acid), waxes (cetylpalmitate), and steroids (cholesterol). Egg lecithin, polysorbates, soybean lecithin, poloxamers, and phosphatidylcholines are commonly used emulsifiers in the production of SLNs. Organic solvents are not required to produce SLNs, whose production yield is high, and scale up is easy than the other type of nanoparticles. Despite these advantages, SLNs production has several limitations, such as pressure-induced drug degradation, gelation, change in the physical state of lipid (formation of supercooled melt), stability problem issues due to the increase in particle size, and drug expulsion (Awasthi et al., 2020). The preparation methods of these nanolipid particles are classified into primary and secondary steps. The primary step describes the preparation of nanolipid particles, and the secondary step describes the drying of synthesized nanolipid particles by lyophilization or freeze-drying to improve the storage stability. Sterilization of SLNs and NLCs is of prime importance as these are commonly administered *via* parental route. Therefore, sterilization is recommended as a secondary step for the production of nanolipid particles.

#### Primary Production Step

The methods for the preparation of the SLNs and NLCs can be classified into (i) methods requiring high energy, (ii) methods requiring low energy, and (iii) methods that utilize the organic solvent.

##### High Energy Method of Preparation

*High-Pressure Homogenization Technique.* This is the most widely used, dependable, and effective technique for the preparation of SLNs and NLCs. In this technique, high-pressure homogenization takes place which pushes the liquid with high pressure (100–2,000 bar) through a few microns gap. Hot homogenization technique and cold homogenization technique are the two methods by which high-pressure homogenization can be achieved.

*Hot Homogenization Technique.* In the hot homogenization technique, the drug is added to the lipid. The temperature is maintained at 5–10°C above the melting point of lipid. The mixture is added by stirring in an aqueous surfactant solution maintained at the same temperature to form a pre-emulsion. The resulting oil dispersed in water (o/w) microemulsion is homogenized and cooled to room temperature to form SLNs (Ekambram and Sathali, 2012). This technique has the advantage of incorporation of lipophilic and insoluble drugs. The limitation of this method is that the loaded drug can be degraded at processing temperature.

*Cold Homogenization Technique.* Cold homogenization technique can diminish the limitations of the hot homogenization technique, including partitioning of lipophobic compounds into an aqueous phase at high temperature, drug degradation at high temperature, and intricacy of the crystallization. The drug is incorporated into melted lipid and cooled rapidly using liquid nitrogen or dry ice (Ekambram and Sathali, 2012).

##### Low Energy Method of Preparation

*Microemulsion Based Technique.* Microemulsions are biphasic systems that are composed of oil and aqueous phases. These are synthesized by stirring a transparent mixture of lipid, co-emulsifier, emulsifier, and water at 65–70°C. The hot microemulsion formed is dispersed in cold water maintained at 2–3°C with stirring to synthesize a suspension of lipid particles. The suspension is filtered and washed with the dispersion medium to obtain final SLNs. Low production yield is the major limitation of this method (Rabinarayan and Suresh, 2010).

*Double Emulsion.* In this method, the drug is dissolved in an aqueous phase followed by emulsification in the melted lipid. A stabilizer is added to stabilize the primary emulsion. This technique is recommended to encapsulate hydrophilic drugs (peptides) and does not require sophisticated equipment (Mahajan et al., 2014). Physical instability like particle growth during storage and broader particle size distribution are the major disadvantages of this technique.

*Membrane Contactor Technique.* In this technique, the liquid phase is pressed through the membrane pores at a temperature above the melting point of lipid to obtain small droplets. The product is cooled at room temperature (25°C) to obtain SLNs. Easy manufacturing and control of particle size by varying process parameters are the major advantages of this technique.

##### Organic Solvent-Based Method of Preparation

*Solvent Emulsification-Evaporation Technique.* In this method, the drug and lipid are dissolved in a non-polar organic solvent (toluene, cyclohexane, dichloromethane, and chloroform). Emulsification is carried out in an aqueous phase using a high-speed homogenizer. The organic solvent is evaporated by stirring at room temperature (25°C) under reduced pressure at 40–60 mbar. Precipitation of lipid leads to the formation of SLNs (Mahajan et al., 2014). Due to the non-thermal processing conditions, this method can be used for the preparation of thermo-labile drug-loaded SLNs. The use of an organic solvent is the major limitation of this method which may interact with drug molecules and limit lipid solubility in the organic phase.

*Solvent Emulsification-Diffusion Technique.* In this technique, the emulsification of lipid matrix in water is carried out under reduced pressure. In an aqueous medium, the lipid gets precipitated and nanoparticle dispersion is obtained. This technique yields the particles with average diameters of 30–100 nm (Muller et al., 2000). The solvent used (e.g., isopropyl acetate, butyl lactate, benzyl alcohol, ethyl acetate, and methyl acetate) must be partially miscible in water. This technique does not require heat and hence can be used for thermo-labile drugs (Trotta et al., 2003).

*Solvent Injection Technique.* This is a new approach to produce SLNs and NLCs, where the solid lipid is dissolved in a water-miscible solvent mixture or water-miscible solvent (ethanol, isopropanol, and acetone,). The organic phase is injected through an injection needle at a constant rate into an aqueous phase with or without surfactant under constant stirring and the dispersion is filtered. The use of pharmacologically acceptable organic solvent, easy handling, and fast production process without using technically sophisticated equipment are the major advantages of this method.

All these preparation techniques have their own advantages and disadvantages (Table 1). The hot homogenization and cold homogenization methods are the most common and widely used methods for the preparation of SLNs and NLCs. The emulsion method requires simple machines and less energy to produce SLNs and NLCs. However, instability during storage is the major limitation associated with these methods. In the organic solvent-based techniques, no sophisticated equipment is used for the preparation of lipid nanoparticles. However, the organic solvent may get interact with the loaded drug molecule.

**Table 1:** Methods for the preparation of SLNs and NLCs.

**Methods**		**Advantages**	**Disadvantages**
High energy methods	High pressure homogenization (hot homogenization and cold homogenization**)**	Widely used method for the preparation of SLNs and NLCs Hot method is used to heat stable materials whereas cold method is used for thermo-labile drugs	Polydisperse distributions and unproven scalability
	Microwave-assisted	This method is not frequently used	–
	Ultrasonication	Reduced shear stress and less time consuming	Contamination of metals may occur
	Supercritical fluid technique	No solvent is used as CO_2_ acts as a solvent Particles are obtained as dry powder	Very expensive method
Low energy methods	Microemulsion-based technique	No sophisticated machines are required for the preparation of microemulsion Less energy is required	Highly diluted dispersions of particles are obtained
	Double emulsion	Production of SLN-containing peptides and hydrophilic active ingredients	Particle growth will occur on storage
	Membrane contactor technique	Applicable for large-scale production	–
	Phase Inversion technique	Suitable for thermo-labile drugs	Tedious process
Organic solvent-based methods	Solvent emulsification-diffusion technique	Most used method for the preparation of the NLCs	Solvent may interact with the drug molecule
	Solvent emulsification-evaporation technique	Most used method for the preparation of SLNs	Solvent may interact with the drug molecule
	Solvent injection technique	Easy handling and fast production process	Solvent may interact with the drug molecule

#### Secondary Production Steps

##### Sterilization

Sterilization is a mandatory process when the formulation is administered parentally (Muller et al., 2008; Yadav et al., 2013). The methods used for the sterilization of SLNs and NLCs are autoclaving (steam sterilization), gamma-irradiation, and filtration. Nanoparticles containing thermostable drugs are sterilized by the steam sterilization method. Steam sterilization at 121°C (15 min) and 110°C (15 min) leads to the aggregation of particles. But it is the preferred method of sterilization as it does not affect mean particle size and zeta potential. Gamma-irradiation is the most widely used alternative sterilization method for thermosensitive drugs. However, irradiation may lead to the chemical degradation of lipids. The filtration-sterilization method can be applied for the particles with a size larger than filter pores. However, this method has not been reported extensively for NLCs.

##### Lyophilization

Lyophilization of the synthesized SLNs and NLCs is important as it helps in maintaining their chemical and physical stability for long-time storage. It is essential especially for the long-term stability for products containing hydrolyzable drugs or a suitable product for peroral administration (Yadav et al., 2013). The conditions used during lyophilization to remove water can promote aggregation of SLNs. However, the aggregation of SLNs can be minimized by using the optimum amount of cryoprotectants (Jose and Netto, 2018).

##### Spray Drying

Spray drying is cheaper and is an alternate method to lyophilization for achieving the chemical and physical stability of the SLNs (Jose and Netto, 2018).

## Characterization of SLNS and NLCS

The characterization of nanocarriers is essential for their clinical applications. The characterization of SLNs and NLCs is challenging because of their very small size and the system is dynamic as compared to the other colloidal carriers. Characterization parameters have a direct impact on the stability and *in vivo* performance of nanoparticles. The SLN and NLC are mainly characterized for particle size, morphology, polydispersity index (PI), zeta potential, percentage drug entrapment efficiency, drug crystallinity, and stability (Üner, 2006).

### Size and Morphology

Accumulation of lipid nanocarriers in the target tissue depends on their physicochemical characteristics including particle size distribution. Successful formulation of safe, stable, and efficient nanocarriers, therefore, requires the preparation of homogenous (monodisperse) populations of nanocarriers of a certain size (Danaei et al., 2018). Normally, laser diffraction (LD) and photon correlation spectroscopy [also called, dynamic light scattering (DLS)] are used to determine the size of the lipid particles. Both techniques are used to assess the size distribution, which is represented by the polydispersity index (PI). DLS gives cumulative information about solution homogeneity and particle size. A single sharp peak indicates the existence of a single and uniform population of scatterers (Ag Seleci et al., 2016). The preferred particle size for NLCs is <400 μm (Salvi and Pawar, 2019). The value of PI ranges from 0.00 (for a perfectly uniform sample in terms of particle size) to 1.00 (for a highly polydisperse sample with multiple particle size distribution). PI values smaller than 0.05 are mainly seen with highly monodisperse standards. PI values bigger than 0.7 indicate a broad particle size distribution. DLS technique is probably not a suitable approach to analyze a sample with broad particle size distribution (Danaei et al., 2018). Other techniques which provide particle size information at sub-micron and nanometric sizes are advanced microscopy techniques, such as scanning electron microscopy (SEM), transmission electron microscopy (TEM), and atomic force microscopy (AFM) (Tamjidi et al., 2013; Gordillo-Galeano and Mora-Huertas, 2018).

### Zeta Potential

It specifies the extent of charge on the surface of the particles in aqueous dispersion which is an important parameter in predicting long-term physical stability of the formulations. Normally, the zeta potential value of lipid nanoparticles is predicted by the electrophoretic mobility and is determined by the photon correlation spectroscopy and LD techniques (Tamjidi et al., 2013; Gordillo-Galeano and Mora-Huertas, 2018). The electrostatically stabilized nanodispersion has zeta potential value >30 mV, in absolute value (Kovačević et al., 2014). The zeta potential is described by the chemical character of particle surface. It also depends on the environment in which the nanoparticles reside, and the sample amount (ASTM International, 2015). In the case of SLNs and NLCs, a minimum zeta potential of higher than −60.0 mV and higher than −30.0 mV is required for excellent and good physical stability, respectively. Formulations containing non-ionic steric stabilizers like polyhydroxy surfactants are expected to have a low zeta potential value. An increased zeta potential value has been reported in SLNs and NLCs with increasing oil content (Kovacevic et al., 2011). Covering of SLNs by a non-ionic surfactant like Tween® tends them to remain stable at a lower zeta potential due to less electrostatic stabilization and greater steric stabilization. Surface coverage of SLNs reduces the electrophoretic mobility of particles and thus lowers the zeta potential (Shah et al., 2014).

### Entrapment Efficiency

The drug entrapment efficiency is the expression of amount of drug entrapped into the carrier with respect to the total amount of drug present in the dispersion. The techniques used for the determination of the entrapment efficiency are the blend of both analytical [UV spectrophotometry or high-performance liquid chromatography (HPLC)] and separation techniques (ultrafiltration, centrifugation, and dialysis). These techniques allow the quantification of the active ingredient. Entrapment efficiency is measured by two major methods, namely direct and indirect methods. In the direct method, the encapsulated drug is directly measured whereas, in the case of indirect method, the amount of unencapsulated drug in the supernatant is measured (Daneshmand et al., 2018). In general, the entrapment efficiency of active ingredients in lipid nanoparticles is above 70%. NLCs are composed of a mixture of liquid and solid lipids. Due to the presence of liquid lipids, an imperfect core is formed in NLCs during the rigidization process. These imperfect cores provide sufficient space for drug accommodation and allow higher drug encapsulation (Salvi and Pawar, 2019). Hence during storage, the drug is not expelled out of core. However, entrapment efficiency of an active molecule into the lipid carriers depends on speed and duration of stirring, the concentration of emulsifier, and surfactant (Daneshmand et al., 2018). Entrapment efficiency is mainly affected by the type, concentration, and crystal structure of lipid. Drug partitioning between melted lipid and aqueous medium also affects the entrapment efficiency of these nanocarriers (Crucho and Barros, 2017). The solubility of a drug in lipids decreases when the molten lipids are cooled; therefore, it is essential to determine the amount of drug mixed with the lipid particles and the amount of drug solubilized in other structures within the formulation. SLNs have higher drug entrapment efficiency for hydrophobic molecules than liposomes (Awasthi et al., 2020).

### Drug Crystallinity

The degree of crystallinity of lipid particles can be established by differential scanning calorimetry (DSC). DSC is an analytical technique in which heat is required to determine the nature of the lipid particles. It is a fast and accurate method for establishing the degree of crystallinity of lipids based on the enthalpy of the lipid. The major disadvantage of DSC is that it is a destructive technique. The non-destructive method which is used to determine the crystallinity of the lipid and to analyze the crystal structure of SLN and NLC is powder X-ray diffractometry (PXRD) (Attama et al., 2008). The use of both solid and liquid lipids is recommended to obtain low degree of crystallinity due to the elaboration of lipid matrix. This provides more spaces to accommodate the therapeutic moieties and minimize drug leakage during polymorphic transitions (da Silva Santos et al., 2019). In case of NLCs, the degree of crystallinity of lipid is decreased with the addition of incompatible solid lipids in the presence of small amounts of oils, whereas in the case of SLNs, the slow drug release can be seen due to high degree of crystallinity (Selvamuthukumar and Velmurugan, 2012).

### *In vitro* Drug Release

The drug release profile from SLNs and NLCs is mainly controlled by biodegradation and diffusion processes. *In vitro* drug release from these nanocarriers is usually estimated by using side-by-side diffusion cells with a biological or artificial membrane-like reverse dialysis sac, ultra-centrifugation, dialysis bag, centrifugal ultra-filtration, and ultra-filtration. The drug release profile is analyzed using a UV spectrophotometer or HPLC (Awasthi et al., 2020).

### Stability

The stability profile of SLNs and NLCs can be studied by determining the mean particle size, size distribution, entrapment efficiency, and drug release profile over storage periods at different temperatures as per the ICH guidelines. The samples are withdrawn at predetermined time intervals and examined for these parameters. The percentage drug entrapment efficiency and drug release profiles are analyzed using a UV spectrophotometer or HPLC (Kovacevic et al., 2011; Shah et al., 2014).

## Scale-up and Stability Issues Associated with Lipid Nanocarriers

The manufacturing of SLNs and NLCs can be easily scaled up and it requires little capital investment as few types of processing equipment are needed to synthesize these particles. However, several stability issues can be linked with the production and scale-up of SLNs. These include polymorphism, phase separation, lipid modification, and storage conditions.

### Polymorphism

It has been proposed that the matrix lipid recrystallization destabilizes the suspension *via* transformation of spherical particles to needle-shaped particles. The particles are converted into needle shape due to the thermodynamic stability and highly ordered β-structure in the lipid matrix. This phenomenon can also be affected by the presence of surfactants, impurities, and stabilizers (Eaton et al., 2017). In the spray drying method, unstable polymorphic forms are formed due to the rapid solvent evaporation. A similar phenomenon has been reported in the spray-congealing method (Salminen et al., 2016). The α-form of triglyceride can be transformed into β-form, which is the most stable form with the high melting point. The unstable form subsequentially transforms into the most stable form during the storage and loses the initial spherical shape of nanoparticles. This forms crystalline aggregates and allows leakage of drugs due to decreased amorphous zones in the carrier matrix (Souto and Muller, 2008).

### Phase Separation

Particle aggregation may lead to the irreversible (coalescence, sedimentation) or reversible (flocculation) phase separation. Gelling of the formulation can occur during storage. Surfactants are used to overcome the aggregation and gelling of formulation which stabilizes NLC suspension by electrostatic repulsion. Cationic or anionic surfactants can increase zeta potential value, and non-ionic surfactants can act as steric stabilizers (Cavalli et al., 1999). The solid forms can overcome the storage stability problems of liquid lipids. This can be achieved by lyophilization or spray-drying of suspension. However, along with this, other formulation parameters must be considered to obtain a re-dispersible powder. Spray-drying causes coalescence of the particles due to the melting of lipid at higher processing temperature. No particle aggregation occurs in freeze-dried SLNs prepared by coacervation method and thus a re-dispersible powder can be obtained. These formulations contain polymeric stabilizers which act as cryoprotectants (Wissing et al., 2004).

### Lipid Modification

The SLNs and NLCs are dynamic systems in which the lipid molecules are thermodynamically unstable. This type of configuration has a higher capability to incorporate drugs. The advantage of higher incorporation rates in unstable modifications is paid off by improved mobility of the drug. During storage, the rearrangement of crystal lattice might form a thermo-dynamically stable configuration which leads to the expulsion of the drug molecules.

### Storage Condition

Proper storage is essential for the stability of SLNs and NLCs. The ambient temperature for the storage of SLNs and NLCs is 4°C. No aggregation or drug loss is observed from the SLNs and NLCs stored at 20°C. However, a prompt increase in particle size has been reported at 50°C. Storage temperature and pH affect the stability of NLCs. At a higher temperature, the particles may form an aggregate due to the breakage of hydrogen bonds between the surfactant at lipid/water interface. A decrease in the zeta potential value of formulations stored at 22°C for a longer duration could be associated with the aggregation and agglomeration of the particles (Haider et al., 2020).

## Drug Encapsulation/Incorporation and Release From SLNS and NLCS

### Drug Encapsulation/Incorporation

The SLNs and NLCs are designed for the inclusion of hydrophilic/hydrophobic drugs. Three models have been proposed for the drug encapsulation: (i) homogenous matrix model, (ii) drug enriched shell-core shell model, and (iii) drug enriched core-core shell model (Muller et al., 2000; Pardeshi et al., 2012; Severino et al., 2012).

#### Homogenous Matrix Model

In this model, the core contains a drug in either amorphous clusters or molecularly dispersed phase. This model is applicable for the encapsulation of highly hydrophobic drugs either by hot homogenization or cold homogenization method (Mishra et al., 2018).

#### Drug Enriched Shell-Core Shell Model

In this model, the drug is present near the shell; hence, the lipid core is free from drug. On cooling the solution, phase separation and lipid precipitation lead to the formation of lipid core free from drug. At the same time, the drug partitions into the leftover liquid-lipid phase and its concentration is enhanced in the outer shell of the lipid core.

#### Drug Enriched Core-Core Shell Model

A drug-enriched core is formed by liquefying a drug in the lipid to its saturation solubility whereby a nanoemulsion is formed. Supersaturation of the drug in lipid melt occurs during the cooling of the nanoemulsion. This leads to the precipitation of a drug before the precipitation of lipid. Further cooling causes the lipid to encompass the precipitated drug, which acts as a membrane for drug encapsulation (Esposito et al., 2018).

### Drug Release

The success of any drug delivery carrier system relies on the release profile of loaded drugs. The drug release from SLNs takes place by degradation, erosion, or diffusion. The mechanism of drug release from SLN matrix depends on lipid and its composition, temperature or surface-active agents, particle size, and pH.

#### Lipid and Its Composition

In SLNs, the bioactive molecules are either distributed in the matrix or present on the surface. These systems show a biphasic release profile with an initial burst release of the surface drug followed by sustained release of drug from the matrix. This indicates that the diffusion of surface drug shows an instantaneous release effect; thereafter the matrix degrades depending upon the lipid composition and the drug is released in a controlled manner.

#### Temperature or Surface-Active Agents

Temperature and surfactants play an important role in controlling the aqueous solubility of drugs. A burst drug release effect has been reported from the SLNs at high temperatures or in the presence of a high concentration of surfactants (Rabinarayan and Suresh, 2010). Therefore, the SLNs should be manufactured at room temperature to prevent burst release and partitioning of the drug in aqueous phase. Hence, the maximum amount of drug is partitioned in lipid phase which leads to a sustained or controlled release of the drug instead of the instant drug from SLNs. The high process temperature may solubilize the drug in an aqueous medium and lead to the accumulation of drug on the outer surface of the lipid matrix (Venkateswarlu and Manjunath, 2004). Rehman et al. demonstrated fast release of 5-fluorouracil (>90%) loaded in SLNs at 39°C due to the melting of lipid. On the other side, only 22–34% of the drug released at 37°C due to the solid lipid core (Rehman et al., 2017).

#### Particle Size

The particle size also affects the drug release from SLNs. Smaller particles have a large effective surface area and hence it provides a quick release of drug in comparison to larger particles.

#### pH

The drug release profile from SLNs is considerably affected by the pH medium. Chen et al. reported pH-sensitive drug release profile of doxorubicin from cholesterol-polyethylene glycol (PEG)-coated SLNs. A fast release profile of doxorubicin was observed at pH 4.7 as compared to pH 7.4. The enhanced rate of drug release at low pH may be due to the decreased electrostatic attractions among the negatively charged lipid core and the positively charged doxorubicin (Chen et al., 2015; Mishra et al., 2018).

## Applications of SLNS and NLCS in Drug Delivery Systems

Several potent synthetic drugs and phytoconstituents cannot be delivered as such or *via* conventional delivery systems due to their absorption problem, low bioavailability, and stability issues. To overcome these limitations, SLNs and NLCs are the choices of drug delivery carrier for improved therapeutic benefits of such molecules (Table 2).

**Table 2:** SLNs/NLCs of natural and synthetic bioactive components administered by different routes.

**Name of the drug**	**Used for**	**Reason for incorporation in the SLN/NLCs**	**Route of administration**	**References**
Quercetin	Anti-obesity	To enhance the bioavailability	Oral	Ganesan et al., 2018
Curcumin	Anti-cancer	Improved antitumor activity and brain targeting *in vitro*	Nasal	Madane and Mahajan, 2016
Triptolide	Anti-cancer activity	Improved absorption and oral bioavailability and reduce the gastric irritation	Oral	Zhang et al., 2013
Puerarin	Anti-hypertensive	Improved absorption and oral bioavailability	Oral	Luo et al., 2011, 2013
Cantharidin	Skin problem	Improved absorption and oral bioavailability.	Oral	Dang and Zhu, 2013
Resveratrol	Anti-obesity	Improved absorption and oral bioavailability	Oral	Neves et al., 2013
Tetrandrine	Calcium channel blocker	Decreases irritation of eye mucous membrane *in vivo*	Ocular	Li et al., 2014
Silymarin	Anti-cancer	Increased absorption and oral bioavailability *in vivo*	Oral	Shangguan et al., 2014
β-Elemene	Anti-cancer	Decreased the irritation and toxicity and enhanced the bioavailability	Oral	Shi et al., 2013
Breviscapine	Anti-hypertensive	Sustained release and protection against the liver enzyme degradation *in vivo*	Oral	Li et al., 2013
Genistein	Anticancer	Enhanced bioavailability	Oral	Kim et al., 2017
Epigallocatechingallate	Anti-obesity effect	Enhanced bioavailability	Oral	Ganesan et al., 2018
Hydroxycitric acid	Cardioprotective	Enhanced bioavailability	Oral	Ganesan et al., 2018
Tripterine	Anticancer	Enhanced bioavailability	Oral	Yuan et al., 2013
Ficusbenjamin	Hepatoprotective	Enhanced bioavailability	Oral	Sharma et al., 2017
Sesamol	Hepatoprotective	Lower irritation	Oral	Sharma et al., 2011
Naringenin	Antioxidant	Increased bioavailability	Pulmonary	Ganesan et al., 2018
Domperidone	Gastro-esophageal reflux	Increased bioavailability by reducing first pass metabolism	Oral	Thatipamula et al., 2011
Lovastatin	Anti-hyperlipidemic	Low bioavailability	Oral	Chen et al., 2010
Methotrexate	Anti-cancer	Site specific targeting	i.v.	
Tamoxifen	Breast cancer	Prolonged the release	i.v.	Murthy, 2005
Mitoxantrone	Breast cancer	Increased bioavailability and reduced toxicity	i.p.	Kuo and Chung, 2011
Rafampicin	Tuberculosis	Improved bioavailability	Nasal	Vila et al., 2004
Isoniazid	Tuberculosis	Improved bioavailability	Nasal	Vila et al., 2004
Pyrazinamide	Tuberculosis	Improved bioavailability	Nasal	Vila et al., 2004
Ciprofloxacin	Antimicrobial	Reduce dose and adverse effects	Oral	Alarifi1 et al., 2020
Nabumetone	Anti-inflammatory	Prolonged the action	Topical	Chaudhari and Ghodake, 2019

### Oral Delivery

The oral route is the most favored route of drug administration as it has numerous advantages over the other route of administration due to the easy administration of drug, accurate dosing, and patient compliance (Khosa et al., 2018). Though this is the most favorable route of administration, all the drugs cannot be administrated *via* this route due to the problems associated with their solubility and poor bioavailability. Therefore, there is an urgent need to develop a novel drug delivery system that can overcome these limitations and elevate therapeutic efficacy by reducing drug dose and its side effects. Lipid nanoparticles, such as SLNs and NLCs offer advantages, such as enhanced solubility, augmented permeability, bioavailability, and better stability by protecting active molecules from various physiological conditions. The enhanced oral bioavailability of drugs administered *via* SLNs and NLCs is due to their enhanced uptake by the lymphatic system in intestinal membrane and avoiding the first pass metabolism (Desai et al., 2012; Lin et al., 2017). The improved absorption of drugs from the orally administered SLNs/NLCs could be due to the increased residence time in the stomach and small intestine. The nanosized SLNs and NLCs have a large surface area and hence gets adhered to the gut wall and increases the absorption of the drug (Zhou et al., 2015).

### Pulmonary Delivery

This is a non-invasive route for drug administration for both local and systemic effects. Pulmonary route has fast absorption of drug molecules due to the increased surface area, enhanced blood supply, and low or no first-pass metabolism. This route has been reported for the administration of several drugs used to treat diseases, such as cancer, acute pain, immune deficiencies, autoimmune diseases, and infections (Weber et al., 2014). These nanocarriers have several benefits, such as uniform drug distribution in the alveoli, enhanced bioavailability, lesser degradation of the released drug, reduced side effects, and better patient compliance (Khosa et al., 2018).

### Topical Delivery

Topical drug delivery is preferred for the treatment of skin diseases. This route has very limited systemic side effects as compared to other routes. The topical route avoids first-pass metabolism and maintains drug concentration for a prolonged duration at the site of application. However, low drug uptake is the major limitation associated with topical drug delivery due to the presence of stratum corneum. During the recent years, lipid nanoparticles, such as SLNs and NLCs have gained considerable attention over conventional topical formulations, such as creams and emulsions due to their sustained release effect, increased permeability, and minimum skin irritant properties. Drugs like podophyllotoxin (Zhao et al., 2016), flurbiprofen (Han et al., 2012), indomethacin (Ricci et al., 2005), and celecoxib (Joshi and Patravale, 2008) encapsulated in SLNs and NLCs have shown better skin permeation, prolonged-release, and decreased skin irritation.

### Ocular Delivery

Physiological barrier of the eye is challenging for the delivery of therapeutic moieties, specifically to the posterior segment of the eye. The ocular retention of bioactive molecules may decrease due to the continuous secretion of tear fluid after the instillation of ophthalmic formulation. Only 5% of the administered drug *via* non-invasive approach remains at the site of administration for a prolonged period. Therefore, invasive methods are sometimes used for the administration of drug which may lead to infection, bleeding, and affects the vision (Gaudana et al., 2010; Patel et al., 2013). Hence, a non-invasive and efficient drug delivery system is required for the ocular administration of therapeutics. SLN/NLC formulations are the choice for ocular drug delivery system due to the improved corneal permeation and increased bioavailability. These formulations are safe, non-invasive, and have good patient compliance. The SLNs and NLCs have more residence time at the site of administration, enhanced therapeutic benefits, and minimum or no local side effects due to their mucoadhesive property. These formulations have been investigated widely for the treatment of ocular disorders, such as inflammation, infections, glaucoma, and disorders affecting the posterior segment of the eye (Sánchez-López et al., 2017).

### Brain Delivery

During the past two decades, a significant increase in the number of neurodegenerative disorders have been reported. Therefore, there is an urgent need to discover new drugs for the treatment of CNS diseases, such as Parkinson's disease, Alzheimer's disease, and brain infections. The main challenge with drug delivery to CNS is that almost 98% of the newly discovered drugs are not able to cross the blood-brain barrier (Beloqui and Solinis, 2016). The nanoparticulate carriers have shown great potential in the delivery of the bioactive molecules to the brain due to their higher retention in the blood-brain capillaries that creates a concentration gradient. This leads to the increased transport of nanoparticles across the brain (Chen et al., 2016). The functional groups on the surfactants, such as PEG and low-density lipoprotein can increase the delivery of therapeutic moieties through the BBB due to the increased binding of drugs with BBB receptors (Gao, 2016). The NLCs are better than SLNs due to their smaller size and high drug loading. However, very limited studies reported NLCs for brain treating (Beloqui and Solinis, 2016). Curcumin has very low bioavailability and high metabolism. Curcumin-loaded NLCs have shown 6.4 times higher plasma concentration and improved brain targeting (Chen et al., 2015). Lim et al. reported 2-fold increase in brain concentration of itraconazole from the synthesized NLCs (Lim et al., 2014). Intravenously administered baicalein-loaded NLCs showed 7.5 and 4.7 times increased accumulation in the cerebral cortex and brain stem, respectively, than its aqueous solution (Tsai et al., 2012).

## Toxicity of Lipid Nanocarriers

Toxicological study of bioactive molecules and excipients used in the formulation is important to impose these formulations for clinical investigations. Unfortunately, limited information on the toxicity profile of nanoparticle-based drug therapies is available (Campos et al., 2020). The lipid nanocarriers that is SLNs and NLCs are ideal for the incorporation of the hydrophilic and lipophilic bioactive compounds. These have many advantages over the other nanocarriers in terms of biocompatibility, protection of bioactive compounds from chemical degradation, site-specific controlled drug delivery, and high drug loading capacity. The other advantages, include ease to scale up and stability during sterilization. No toxicity data is available to date for the SLNs and NLCs. The major components of these nanocarriers are physiological lipids and excipients which are generally recognized as safe (GRAS) (Campos et al., 2020). However, these nanocarriers are prepared for the delivery of bioactive molecules in the human body; hence, before launching a product to the market, their toxicity profile must be studied (Marchan et al., 2012). Though sufficient data regarding the efficiency and quality of SLNs and NLCs is available, limited information is available regarding the safety of these lipid nanoparticles. This is due to the synthesis of these nanocarriers using GRAS excipients. This concept holds good for oral and topical administration, whereas this is not true with the parenteral delivery. Surfactants can activate the immune system. Therefore, *in vitro* toxicity studies, such as cytotoxicity, genotoxicity, and hemocompatibility may provide promising safety data (Doktorovová et al., 2016).

### Cytotoxicity

The biocompatibility of lipid nanoparticles is tested by the determination of cytotoxicity or cell viability. Normally, a cytotoxicity assay is conducted to check the potency of anticancer drugs. In cytotoxicity assay, cell viability is tested as an evidence of formulation efficiency. SLNs and NLCs are used to increase the efficiency of chemotherapeutics by improving the cellular uptake of the drugs. However, these should remain inactive and should not influence the cell viability. Lipids that are used to prepare NLCs and SLNs are tolerated at high doses by different cell lines (Doktorovová et al., 2016). However, most of the cell lines tolerate 1 mg/ml lipid SLNs/NLCs (Doktorovova et al., 2014).

Akanda et al. reported stearic acid and poloxamer 188 containing retinoic acid-loaded SLNs. This formulation was tolerated at a 3 mg/ml dose by androgen-sensitive human prostate adenocarcinoma, LNCaP cells (Akanda et al., 2015). Ghalaei et al. reported cetyl alcohol-containing hyaluronic acid-coated SLNs (stabilized by polysorbate 80 and containing stearylamine). The synthesized SLNs were tolerated at high doses by SK-OV-3 cells (Ghalaei et al., 2014). Cetyl alcohol-based SLNs are stabilized by polyvinyl alcohol and polysorbate 80 are tolerated by MCF-7 cells at doses reaching 2 mg/ml (Parveen et al., 2014).

Surfactants also play an important role in the stabilization of SLNs. Cetyltrimethylammonium bromide (CTAB) is the cationic surfactant that can be used at 1 mg/ml without affecting cell viability. But there are some reports which showed that the cationic surfactants affect the integrity of the cell membrane (Xue et al., 2014). Almeida et al. reported CTAB, glyceroldibehenate, and poloxamer 127 containing SLNs are safe at 1 mg/ml without compromising cell viability (Almeida et al., 2015). Fangueiro et al. reported that the impact on cell viability of Y-79 cells can be controlled by limiting CTAB content to 0.5% in SLNs (Fangueiro et al., 2014).

### Genotoxicity

No DNA damage in A549 cells has been reported by the negatively charged SLNs. The study reported that there was no DNA fractionation in gel electrophoresis (Dolatabadi et al., 2014; Bhushan et al., 2016). Brugé et al. further supported these results using a more sensitive comet assay method where the scoring of DNA fluorescence intensity in comet tail (vs. comet head) is an indicator of DNA strand break occurrence. The study reported that there was no increase in the percentage of tail intensity for blank and Q10-loaded NLCs in human dermal fibroblasts. The results indicated that the synthesized NLCs had no genotoxicity (Brugè et al., 2013). Eskandani et al. reported that the DNA damage on acetyl shikonin-containing SLNs caused an increase in comet formation in A549 cells. The free drug had DNA damage effect which was further increased when the drug was encapsulated in SLNs. However, at the concentration selected for genotoxicity testing, the drug and drug-loaded SLNs decreased the cell viability below 50% (Eskandani, 2014). Hence the negatively charged SLNs do not have genotoxicity.

### Hemotoxicity

Hemolysis assay is performed to determine the damage of red blood cells when a foreign material or a drug is injected through an intravenous route. Intravenously administered SLNs/NLCs do not damage RBCs. Lakkadwala et al. reported that the SLNs composed of glycerol monostearate and polysorbate 80 have limited hemolysis even at 1 mg/ml dose (Lakkadwala et al., 2014). Chemotherapeutic drug-loaded SLNs coated with hyaluronic acid containing a cationic compound (stearic acetate) had low hemolytic activity (Negi et al., 2014). Doxorubicin-loaded cationic SLNs decreased hemolysis. This effect was further enhanced when SLNs were coated with galactose (Jain et al., 2015).

## Recent advances

In the past, nanoparticles were commonly administered through the parenteral route; however, now scientists are exploring various other dosage forms containing nanocarriers. Some of the patents are shown in Table 3. Formulations based on SLNs and NLCs have been entered into the clinical phases (Table 4). However, few SLNs and NLCs based products are under clinical investigations or are available commercially. Most of the SLN-based products are in Phases 1 and 2 of the clinical trials and few products are in the pipeline. We can expect many more products to enter the clinical phase leading to commercial success in the near future, which shows that the future of these nanocarriers is promising and there is scope to explore more in this area.

**Table 3:** Recent patents on SLNs and NLCs.

**Patent/Publication number**	**Status**	**Publication/grant date**	**Assignee**	**Title**	**Drugs disclosed**	**Dosage Form**
**Solid lipid nanoparticles**						
CN110585171A	Active	December 12, 2019	Wuhan Burunnao Medical Technology Co., Ltd.	Temozolomide solid lipid nanoparticle and preparation method thereof	Temozolomide	Oral administration, intravenous injection, pulmonary inhalation, local administration, and the like
US20200197360A1	Active	June 25, 2020	SRM Institute of Science and Technology	Dispersion of formononetin solid lipid nanoparticles and process for its preparation	Formononetin	Injection
WO2020144377A1	Active	July 16, 2020	The Queen's University of Belfast	Solvent and water-free lipid-based nanoparticles and their methods of manufacturing	Felodipine, Naproxen, and Ketoconazole	Reconstituted prior to infusion or injection
WO2020109989A1	Active	Jun 04, 2020	Panjab University Chandigarh	Solid lipid nanoparticles of curcumin	Curcumin	Gel, hydrogel, organogel, syrup, paste, cream, facewash, mouthwash, oral rinse, ointment, liquid ampoule, dispersion, aerosol spray, powder, orthotic aid, liquid oral, FDM facemask, implant, tablet, lozenges, capsules, suppositories, pessaries, patch, and gummies
KR20200085529A	Active	Jul 15, 2020	Chungbuk National University Industry-Academic Cooperation Foundation	Solid lipid nanoparticles for skin permeation and composition for drug delivery comprising the same	Tacrolimus, dexpanterol, and allantoyl	–
WO2019206738A1	Active	Oct 31, 2019	Chr. Hansen Natural Colors A/S, Københavns Universitet	Pigment-loaded solid lipid nanoparticles	Naturally occurring oil-soluble pigments, such as carotenoids or chlorophylls. Carotenoids include alpha- carotene, beta-carotene, lycopene, lutein, bixin, and norbixin	Used in food products and beverages
WO2020028916A1	Active	February 06, 2020	University of Mississippi	Amphotericin loaded pegylated lipid nanoparticles and methods of use	Amphotericin	Injection
WO2020053609A1	Active	March 19, 2020	Lead Biotherapeutics Ltd.	Mucoadhesive dispersion. Nanoparticle system and method for the production of the same	Mometasonefuroate Xylometazoline Loratidine	Nasal mucoadhesive
CN110585121A	Active	December 20, 2019	Wuhan Burunnao Medical Technology Co., Ltd.	Temozolomide nuclear magnetic resonance visual injectable hydrogel, preparation method and application	Temozolomide	Injectable hydrogel for diagnostic purpose
US20200114019A1	Active	April 16, 2020	National Yang Ming University	The pH-sensitive lipid nanoparticles for encapsulation of anticancer drugs and microrna and use thereof	Anticancer Drugs: Irinotecan, oxaliplatin, epirubicin, doxorubicin, afatinib, and docetaxel	Injection
**Nano structure lipid carriers**						
CN108853056A	Active	Nov 23, 2018	Tianjin University of Traditional Chinese Medicine	A kind of modification of folate-targeted carries doxorubicin hydrochloride and gambogic acid nano structured lipid carrier preparation and preparation method thereof altogether	Doxorubicin hydrochloride and gambogicacid	–
WO2019226186A1	Active	November 28, 2019	U.S. Nutraceuticals, Llc D/B/A Valensa International	Composition and method to alleviate joint pain using hyaluronic acid and eggshell membrane components	Hyaluronic acid	Injection
US20200010530A1	Active	January 09, 2020	Tianxin Wang	Methods and reagents to treat autoimmune diseases and allergy	Peptide-MHC I complex and peptide-MHC II complex	Subcutaneous or intravenous injection
CN108685875A	Active	Oct 23, 2018	China Pharmaceutical University	A kind of natural nano grain-pharmaceutical composition of anti-Alzheimer's disease and its preparation method and application	Cholesteryl ester, phosphatidyl choline, triglycerides, ceramide, and gangliosides	Injection
WO2019191780A9	Active	Jun 18, 2020	Arcturus Therapeutics, Inc.	Lipid particles for nucleic acid delivery	Antibody or an immunogenic peptide	Subcutaneous injection, intradermal injection, or intramuscular injection

**Table 4:** SLNs and NLCs in clinical trials.

**Clinical trial number**	**Status**	**Title**	**Drug**	**Dosage Form**	**Completion date**	**Sponsor**	**Source/Reference**
**Solid lipid nanoparticles**							
NCT03823040	Phase 1 completed	Clinical assessment of oxiconazole nitrate solid lipid nanoparticles loaded gel	Oxiconazole nitrate	Drug-loaded SLNs-based gel	December 30, 2018	Minia University	https://clinicaltrials.gov/ct2/show/NCT03823040?term=Solid+lipid+nanoparticlesanddraw=2andrank=1
NCT04035525	Recruiting	Pharmacokinetic study on three formulations of coenzyme Q10 with different carriers	Dietary supplements	–	August 31, 2020	Mélanie Plourde	https://clinicaltrials.gov/ct2/show/NCT04035525?term=Solid+lipid+nanoparticlesanddraw=2andrank=2
NCT02110563	Terminated (sponsor decision)	Phase I, multicenter, dose escalation study of DCR-MYC in patients with solid tumors, multiple myeloma, or lymphoma	DCR-MYC	IV infusion	November 3, 2016	Dicerna Pharmaceuticals, Inc.	https://clinicaltrials.gov/ct2/show/record/NCT02110563?term=Solid+lipid+nanoparticlesanddraw=2andrank=3
NCT03739931	Recruiting	Dose escalation study of mRNA-2752 for intratumoral injection to patients with advanced malignancies	mRNA-2752 mRNA-2752 + Durvalumab	Intratumoral injection/intravenous	July 2021	ModernaTX, Inc. AstraZeneca	https://clinicaltrials.gov/ct2/show/NCT03739931?term=Solid+lipid+nanoparticlesanddraw=2andrank=4
NCT03323398	Recruiting phase 1 and 2 for ovarian cancer	Dose escalation and efficacy study of mRNA 2416 for intratumoral injection alone and in combination with Durvalumab for patients with advanced malignancies	mRNA-2416 Durvalumab	Intratumoral injection/intravenous	March 2022	Moderna TX, Inc.	https://clinicaltrials.gov/ct2/show/NCT03323398?term=Solid+lipid+nanoparticlesanddraw=2andrank=5
NCT02314052	Terminated	Phase Ib/2, multicenter, dose escalation study of DCR-MYC in patients with hepatocellular carcinoma	DCR-MYC for hepatocellular carcinoma	Intravenous (IV) infusion	October 11, 2016	Dicerna Pharmaceuticals, Inc.	https://clinicaltrials.gov/ct2/show/NCT02314052?term=Solid+lipid+nanoparticlesanddraw=2andrank=6
**Nano structure lipid carriers**							
NCT01436123	Terminated (The study was terminated under the political pressure of the Federal Security Service of the Russian Federation (FSB) and the Russian Society of Cardiology)	Plasmonic photothermal and stem cell therapy of atherosclerosis vs. stenting (NANOM PCI)	Everolimus	Stenting and micro-infusion of NP device: implantation of everolimus-eluting stent	October 2012	Ural Medical University	https://clinicaltrials.gov/ct2/show/NCT01436123?term=Nano+structure+Lipid+Carriersanddraw=2andrank=1

## Conclusion and Future Prospective

SLNs and NLCs are nanocarriers that provide benefits in the administration and delivery of bioactive molecules. SLNs and NLCs have been reported as a good option for targeted drug delivery. The present review discusses the recent advances in SLNs and NLCs, including technological progress in the delivery of old and new drugs, process of preparing the same, and their characterization methods. SLNs and NLCs as drug carriers offer benefits in the administration and delivery of both natural and synthetic bioactive molecules. Despite the progress in the field of lipid nanoparticle research, there is still a long way to go before achieving the clinical success of lipid nanoparticle formulations. Though a lot of research and studies are done on the fabrication, storage, and toxicity of the SLNs and NLCs, still some of the problems persist. The scale-up issues, long-term stability issues upon storage, and toxicity are the areas of concern in the development of lipid nanoparticles. The science of SLNs and NLCs is currently among the most appealing areas of research. A lot of research in this field in the last 5 years has already led to the filling of <2,000 patents and completion of few clinical trials across the world. Thus, nanotechnology is expected to introduce new vistas in biomedical science utilizing the advantage of its small size.

## Author Contributions

All authors listed have made a substantial, direct and intellectual contribution to the work, and approved it for publication.

## Conflict of Interest

The authors declare that the research was conducted in the absence of any commercial or financial relationships that could be construed as a potential conflict of interest.
